# Challenges to food security in a changing environment: a community-based participatory research study in Casamance, Senegal (West Africa)

**DOI:** 10.3389/fpubh.2026.1774424

**Published:** 2026-03-31

**Authors:** Noah Barclay-Derman, Seckou Badji, Jacques F. Sambou, Sarah Drummond, André Sadio, Cherif Gueye, Anaïs Senghor, Baboucar Diatta, Jean Phillippe Diatta, Noël Magloire Manga, Sarah Koch, Geoffrey S. Gottlieb, Boubacar Kande, Peter M. Rabinowitz, Moussa Seydi, Noelle A. Benzekri

**Affiliations:** 1 Department of Biomedical and Health Sciences, University of Vermont, Burlington, VT, United States; 2 Development in Gardening, Atlanta, GA, United States; 3 Centre de Santé de Ziguinchor, Ziguinchor, Senegal; 4 Hôpital de la Paix, Ziguinchor, Senegal; 5 Department of Medicine, Division of Allergy & Infectious Diseases, and the Center for Emerging and Re-Emerging Infectious Diseases (CERID) University of Washington, Seattle, WA, United States; 6 Department of Global Health, University of Washington, Seattle, WA, United States; 7 Center for One Health Research, Department of Environmental and Occupational Health Sciences, University of Washington, Seattle, WA, United States; 8 Services des Maladies Infectieuses et Tropicales, Center Hospitalier National Universitaire de Fann, Dakar, Senegal; 9 School of Public Health, University of Washington, Seattle, WA, United States

**Keywords:** climate change, food insecurity, community-based participatory research, agroecology smallholder farming, Senegal, Casamance, mixed-methods

## Abstract

**Introduction:**

Environmental change is accelerating across West Africa, placing increasing pressure on communities whose livelihoods depend on smallholder and subsistence farming. In Senegal's Casamance region, shifting rainfall patterns, rising temperatures, soil salinization, and deforestation are undermining food production and altering culturally significant landscapes, including sacred forests. While ecological changes are well documented, fewer studies examine how local communities interpret these transformations and how these perceptions relate to food security.

**Methods:**

This study used a community-based participatory research design to examine how farmers in Basse-Casamance perceive environmental change, how these perceived changes affect food security and livelihoods, and what locally identified strategies emerge in response. Mixed methods were employed, including focus group discussions, in-depth interviews, and semi-structured surveys with 234 agricultural households across 13 communities.

**Results:**

Qualitative analysis identified six major themes: the destruction of forests; decreasing and irregular rainfall; declining river and marine health; loss and degradation of farmland; the abandonment of land due to conflict; and locally proposed solutions such as reforestation, strengthened environmental governance, and support for sustainable farming practices. Quantitative results showed high levels of food insecurity, with 86% of participating households reporting some level of food access challenges. All participants reported that, based on their experience, the environment in the Casamance is changing, and 100% of participants reported that the changes in the environment are contributing to food insecurity.

**Discussion/Conclusion:**

Community observations closely align with regional climate and ecological data, demonstrating that experiential and place-based knowledge provide an essential complement to scientific assessments. Findings highlight the interconnected ecological, social, and spiritual dimensions of environmental change. Participants emphasized that meaningful responses require upstream, system-level interventions, including policy reform, stronger governmental engagement, and support for agroecological and community-governed food systems. Such strategies are essential for strengthening resilience in the face of accelerating climate and environmental pressures. Environmental change in the Casamance is multifaceted, deeply felt, and closely tied to food security, cultural identity, and land stewardship. Community identified solutions included elevating community knowledge, protecting culturally significant landscapes, and prioritizing policy-level action that supports sustainable, women-led, and locally grounded food systems.

## Introduction

Climate change poses a significant and growing threat to food security in West Africa, creating pressures that communities feel with increasing immediacy. Rising temperatures and increasingly erratic rainfall have already begun to reduce agricultural productivity and weaken the land and water systems that rural households depend on for their livelihoods (1–4). According to the most recent *State of Food Security and Nutrition in the World* (SOFI) report, an estimated 14.6% of the population in West Africa is undernourished, a proportion that has risen in recent years (5). This trend is particularly troubling given that many low- and middle-income countries (LMICs) like Senegal, contribute minimally to global greenhouse gas emissions yet experience the most severe consequences of climate change ranging from prolonged droughts, shifting rainfall patterns to broader environmental degradation that directly affects food availability and access (6, 7).

As a result, climate change and its far-reaching impacts on food systems have become a major concern at local, regional, and international levels (1, 8, 9). While a substantial and growing important body of research has examined the environmental impacts of climate change in West Africa looking at altered precipitation patterns, increasing temperatures, and declining agricultural yields there has been less attention paid to how families and subsistence farmers in West Africa perceive and interpret these changes themselves (10–12). This gap is significant, particularly because these communities are among those most directly affected by climate variability and environmental stress (13). Despite their central role in local food systems and their deep, place-based ecological knowledge, relatively little research explores farmers' experiential understanding, emotional responses, or interpretations of climate-related phenomena and how these shape their food security strategies (14). Community-based participatory approaches are therefore essential for understanding the lived realities, the decision making, and the local meanings that quantitative indicators cannot fully capture.

### Casamance, changing environment, and food security background

In Senegal, roughly one third of the population is employed in agriculture, with even higher dependence in the southern regions (15). In the Casamance, approximately 71% of households identify agriculture as their primary activity, often combining crop production, livestock, fishing, and market gardening (16). This dependence has bidirectional implications for food security where small-scale family farming produces an estimated 80% of Africa's food supply yet farmers and their families make up the largest share of those who experience undernutrition (13, 17–19). Environmental change is intensifying these pressures. A recent study has shown a clear warming trend across Senegal with average national temperatures rising approximately +0.73 °C between 1981 and 2020 and significant warming documented in the Casamance (12). The study also found increasing rainfall variability and more intense precipitation events that complicate planting calendars, heighten flooding risks, and reduces predictability for rain-fed farming. In Casamance, these shifting rainfall patterns and salt water intrusion have also lowered rice productivity, leading to reduced yields and abandonment of marginal lowland fields (10, 11, 20, 21).

These environmental disruptions not only impact agricultural production but also endanger cultural landscapes including sacred forests which are forests protected as spiritual, ancestral, and ritual spaces across West Africa (22, 23). Among the communities in the Basse-Casamance, sacred forests remain central to social and spiritual life, while also playing ecological roles in buffering salinization and seawater intrusion. Environmental changes not only threaten food security but also the cultural and spiritual institutions that sustain community resilience.

This is all under the backdrop of the Casamance conflict, a four-decade-long conflict between the Movement of Democratic Forces of Casamance (MFDC) and the Government of Senegal (24). Despite the signing of a peace agreement in 2022, the conflict has had enduring effects on rural livelihoods, including population displacement, restricted access to land, and persistent constraints on agricultural production with around 80% of farmlands rendered unusable by landmines, all of which intersect with and exacerbate environmental pressures on food system (25).

## Methods

Based on community-based participatory research (CBPR) principles, we conducted a multiple-phase study using the mixed methods participatory-social justice study design to explore how communities perceive these environmental changes and how these shifts affect food security in the Basse-Casamance Region (26). This collaborative approach is particularly important given the region's history of marginalization, conflict, and ecological disruption factors that continually shape local health and livelihoods. The region's communities possess extensive experiential and ecological knowledge from their long-term interaction with their environment, making their insights critical to understanding the shifting conditions, such as soil salinization, variable rainfall, and deforestation. Employing a participatory approach enables the integration of this local knowledge with scientific inquiry, generating a nuanced understanding of how environmental and social dynamics intersect to shape local food systems.

Using the mixed methods participatory-social justice study design, our study began with an exploratory phase followed by a convergent phase (26).

### Phase 1

Focus group discussions and in-depth interviews with community members, stakeholders, and key informants were conducted to explore perceptions, beliefs, and experiences regarding climate change and changes in the environment, and to understand the process by which these changes impact food security and influence behaviors. We conducted exploratory focus group discussions and in-depth interviews with 42 community members, stakeholders, and key informants. The themes that emerged from the exploratory focus group discussions and in-depth interviews contributed to the development of an interview guide. This interview guide was subsequently used to collect both qualitative and quantitative data from community members in the Basse-Casamance region.

### Phase 2

Interviews were conducted with 234 participants, located in 13 communities across an area of approximately 1,100 km^2^ in the Basse-Casamance region. Participants were community members ≥18 years of age with experience practicing agriculture in the region. Participants were selected using stratified purposive sampling based upon geographic location and community of residence (27). All participants provided informed consent. Interviews were conducted in the participant's preferred language, which was predominantly Diola, Wolof, or French. Responses were recorded using detailed notes that were taken throughout the interviews.

The interview guide included both qualitative and quantitative questions, including exploratory open-ended questions and structured closed-ended questions. The Household Food Insecurity Access Scale (HFIAS) was used to measure food insecurity. The 9-item HFIAS was developed by the USAID Food and Nutrition Technical Assistance project to assess household food insecurity across different cultural contexts. It provides a categorical indicator of food insecurity status on a scale of 1–4, with 1 being not food insecure, 2 being mildly food insecure, 3 being moderately food insecure, and 4 being severely food insecure.

Quantitative data were entered into a spreadsheet and uploaded into SPSS Statistics version 29 (IBM) for analysis. Descriptive analysis was performed for all variables. Missing data were excluded from the analysis. Qualitative data, including detailed interview notes, were uploaded into ATLAS.ti version 23.4 (ATLAS.ti Scientific Software Development) and analyzed using thematic analysis and inductive coding.

## Results

### Participant characteristics

Among participants, 134 (57%) were located in rural communities and 100 (43%) were located in Ziguinchor, the semi-urban capital of the region (Table 1). The majority of participants were female (81%) and the median age was 44 years (IQR 36–50; range 20–75). All participants practiced agriculture. Approximately one third (32%) had never received a formal education, 27% had attended primary school, 26% had attended secondary school, and 15% had attended university. The majority of participants (86%) were food insecure (14% were mildly food insecure, 27% were moderately food insecure, and 45% were severely food insecure).

**Table 1:** Participant characteristics, N = 234.

Characteristic	*n* (%)
**Age, median years (IQR;range)**	44 (36–50; 20–75)
Sex	
Female	189 (81)
Educational level	
No formal education	74 (32)
Primary school	63 (27)
Secondary school	61 (26)
University	34 (15)
Food insecurity status	
Food insecure (any level)	195 (86)
Mildly food insecure	31 (14)
Moderately food insecure	62 (27)
Severely food insecure	102 (45)

All participants reported that, based on their experience, the environment in the Casamance is changing, and 100% of participants reported that the changes in the environment are contributing to food insecurity (Table 2). The unanimous reporting of environmental change suggests it is not merely perception but a collective lived experience of systemic ecological transformation.

**Table 2:** Participant perceptions of environmental change and food security *N* = 234.

Question	Yes (%)
Perceptions of environmental change	
Based on your experience, is the environment in the Casamance changing?	100
Has the rainy season changed?	97.0
Is the forest changing?	98.7
Is there deforestation?	97.4
According to your experience, do people cut down trees?	99.1
Perceived impacts on environment and livelihoods	
Are environmental changes impacting agriculture?	99.6
Are environmental changes impacting soil quality?	99.1
Are environmental changes impacting access to water?	94.0
Are environmental changes impacting the river?	97.0
Are environmental changes impacting the sea?	96.9
Are environmental changes impacting fishing?	98.3
Social and cultural impacts	
Are environmental changes impacting traditional beliefs and practices?	86.8
Do traditional beliefs and practices impact the environment?	69.9
Are environmental changes impacting people's practices and behaviors?	94.3
Are the changes in the environment having an impact on people's practices and behavior?	96.9
Do the changes in the environment have the same impact on men and women?	68.9
Are people harming the environment?	98.7
Can people help the environment?	100
Should people help the environment?	100
Conflict, population, and food security	
Has the conflict impacted food security?	99.1
Has population displacement impacted food security?	97.8
Is there population growth?	96.9
Does population growth impact food security?	94.7
Community strategies	
Are there strategies already in place to help the environment?	85.4
Are there strategies already in place to improve food security?	90.1

Six themes emerged from analysis of the qualitative data: (1) destruction of the forest, (2) absence of rain, (3) poor health of the river and the sea, (4) loss of the land, (5) abandonment of the land, and (6) community-proposed solutions.

### Theme 1. Destruction of the forest

Destruction of the forest due to **deforestation** was the central concept discussed by the vast majority of participants and was considered the core consequence of environmental change that was related to all other aspects and impacts. Logging, forest fires, and human habitat expansion were considered the primary causes of deforestation. Destruction of the forest was considered a major cause of decreased rainfall as seen through two worldviews: (1) large trees and forests are believed to capture precipitation and regulate climate, and (2) according to traditional belief systems, the forest and ancient trees are sacred and are the home of ancestors, spirits, genies, and powerful mystical beings that regulate rainfall.

Destruction of the forest was also seen to contribute to food insecurity through multiple direct pathways, including the loss of trees and plants that are food sources and the loss of bushmeat due to habitat destruction. Similarly, destruction of the mangrove forests has led to habitat loss for fish and other animals that are important sources of food. An additional emergent concept was the impact of deforestation on the practice of traditional medicine, as medicinal plants are no longer available or are increasingly rare. Importantly, destruction of the forest was considered a major threat to traditional beliefs, customs, and practices, and as such, poses an existential threat to traditional identity.

### Theme 2. Absence of rain

Changes in the rainy season, specifically **decreased rainfall** and a shorter season, were a dominant concept. The changed rainy season was seen as a major contributor to decreased agricultural production and **decreased harvests**. This was mediated by decreased availability of water for crops, decreased direct irrigation from rainfall, decreased durability of wells, and changes in accessibility of the water table. Decreased rainfall was also considered a cause of increased **salinity** of the soil, rendering agricultural lands unusable or limiting yields, and increased **salinity** of the river, impacting the health of fish and other aquatic animals.

### Theme 3. Poor health of the river and the sea

Changes in fish populations of the river and the sea, including fewer fish overall and the disappearance of large fish, were another dominant concept. This was primarily thought to be the result of abusive fishing practices, industrialized fishing, **pollution**, and inadequate rainfall. In a region where fish have traditionally been a major food source, this was seen to be a major threat to both the food security and food sovereignty of the community.

### Theme 4. Loss of the land

A recurring concept was the loss of land available for food production as a result of both environmental and social factors. This included “impoverishment of the land” or the loss of land available for agriculture due to decreased quality and degradation of the soil. The major factors thought to be contributing to the decreased quality of the soil were **pollution** from toxic chemicals, and increased salinity. This concept also included the “loss of land” available for agriculture and livestock, and encroachment on forested land and wildlife habitat, as a result of population growth.

### Theme 5. Abandonment of the land

The impacts of the decades-long **civil conflict** in the Casamance resonated in the concept of “abandonment of the land”. As a result of the conflict and the widespread use of landmines, there was an exodus from rural zones and forced abandonment of farmland and livestock. This **displacement** has had a lasting impact on agricultural production and food security in the region.

### Theme 6. Community-proposed solutions

Strategies proposed by community members to strengthen resilience in the face of increasing food insecurity and environmental change included, campaigns to halt deforestation and increase awareness of activities that harm the environment, providing training and concrete skills in sustainable agriculture, implementing reforestation initiatives, and reinforcing and expanding the mandate of government environmental agencies (such as the Ministère de l‘Environnement et du Développement Durable and l'Agence des Eaux, Forêts, Chasse et de la Conservation des Sols) to enhance environmental protection and regulation.

## Discussion

The themes that emerged from this study, forest degradation and deforestation, shifting rainfall patterns, soil salinization, and the loss of arable land, are not only central to participants lived experiences but are also strongly supported by other research studies and broader scientific evidence (10, 12, 21, 28–30). The community members‘ descriptions of environmental change align closely with documented regional trends, confirming that their observations are accurate reflections of ongoing climatic and ecological transformations. This convergence between empirical data and participants' accounts underscores that smallholder and subsistence farmers possess deep, place-based knowledge developed through daily interaction with their land. Their insights are therefore not merely anecdotal but constitute a critical source of understanding that can complement and enrich scientific assessments. Recognizing the validity and value of this experiential indigenous knowledge is essential for climate adaptation strategies and food security programs. The discussions below are based on responding to the community's identified themes.

### Agriculture, deforestation, gender, and stewardship

In the Casamance Region and many other parts of Senegal and West Africa, there is a deep connection between forests (many considered sacred) conservation and agricultural practices (31). The relationship between the two shapes how land is used, how farming decisions are made, and how communities, especially women leaders, understand their responsibilities to the environment (32). For many households in the Casamance, subsistence farming is inseparable from a spiritual ethic of care for the land. They also see that the sacred forests supply medicinal plants, regulate water, and protect soil fertility, creating a reciprocal relationship in which humans and ancestors jointly maintain the ecological balance that is needed for rice cultivation, horticulture, and tree-crop production (33). Oftentimes, the forests are positioned near fields or along the boundaries of agricultural zones, serving not only as spiritual sanctuaries but also as ecological buffers. So when deforestation occurs, especially when it affects ancient trees and sacred forests, the consequences extend beyond just ecological loss but also disrupt spiritual traditions, social cohesion, and even local food security (31).

This is especially true for women, whose labor and spiritual authority are central in this landscape (34). In many communities, women serve as custodians of sacred sites, holders of ecological knowledge, as well as primary cultivators of household food crops (35). Their understanding of soil, seasonal cycles, and plant varieties is often framed through a worldview in which the ancestors guide, warn, and bless them creating a moral dimension to food production. Therefore, when considering conservation and food security, it is essential to recognize the intersection of women's leadership, ancestral knowledge, environmental stewardship, and food cultivation. Doing so ensures that farming and conservation are not framed as just economic activities but understood as culturally, socially, and spiritually embedded practices.

### Agroecology and upstream approaches

The interrelationship between agriculture and culture identified by the community is part of the reason why agroecology has gained considerable momentum in West Africa and particularly in Senegal (36, 37). Agroecology frames farming not simply as an economic enterprise, but as an ecological, social, and political practice that links food production to biodiversity conservation and community well-being. Agroecology's potential to strengthen livelihoods and ecosystems is particularly relevant in regions where smallholder, subsistence, and women-led farming predominate, like in the Casamance Region. There have been several recent case studies across West Africa and Senegal that demonstrate the link between agroecology, food security, and conservation from agroforestry and wetland restoration to farmer-managed seed systems that simultaneously reinforce environmental protection and maintain or enhance food security (38, 39).

The participants in the study clearly understood the urgent environmental threats in the Casamance and they emphasized that addressing these challenges cannot be done individually. They highlighted the need for structural and policy-level action, including stronger government engagement in environmental protection, better enforcement of land and forest management rules, and expanded mandates and resources for environmental and agricultural agencies. Meaningful agroecological transitions require government-led, system-wide interventions. There have been several important examples of systems-level approaches that can shape agriculture rather than placing responsibility solely on individual farmers. These include policy reforms that protect forests, wetlands, and community-managed lands; strengthen public agricultural research and extension promoting farmer-led innovation; securing land tenure rights, particularly for women; and creating enabling economic and institutional environments, such as local food procurement policies, ecological subsidies, and biodiversity-protective regulations (40–44).

### Resilience and local food systems

Under the backdrop of the changing ecological environment, there is also a growing number of natural disasters and infectious disease outbreaks linked to climate change (45). In the Casamance region, despite it being largely agrarian, many communities depend on produce imported from Dakar. During Covid-19, pandemic-related travel restrictions halted boat transport, markets thinned out, and food prices soared, yet the farmers who managed their own land, especially those using agroecological or diversified horticultural methods, were not only more food-secure themselves, but also helped buffer the broader community against shortages (46). Such patterns mirror broader findings where smallholder farming systems that rely on local production and collective structures often show enhanced adaptive capacity and food system resilience during crises (39).

From a systems perspective, resilience involves sustaining food security amid uncertainty, maintaining social-ecological balance, and enabling adaptive practices (47). In the context of conflict, natural disaster or pandemic disruption, households embedded in local networks tend to fare better, because they can combine strategies: diversified production, shared resources, and mutual aid (48, 49). Furthermore, agroecological practices like intercropping, agroforestry, and organic soil management promote ecological stability and resource efficiency, directly contributing to resilient food systems (36).

So, as we think about food insecurity, changing environments, and the people who are most impacted, aligning programs with the communities' recognized solutions is imperative. Within that community, it is also important to recognize who leads what roles; in the Casamance, women play a central role in horticulture and seed management. Their leadership in local food production is not just a matter of equity, but a resilience strategy. When women are empowered to steward land, cultivate biodiverse gardens, and manage seeds, the whole community's capacity to withstand climatic or pandemic shock improves (50).

## Limitations

This study has several limitations. The sample was limited to select communities within the Casamance region, which may constrain the generalizability of findings to other parts of Senegal. Additionally, while qualitative methods provided rich contextual insight, they do rely on the self-reported experiences of the participants that may introduce recall or social desirability bias. Finally, because environmental and agricultural conditions are rapidly evolving, the findings represent a snapshot in time.

## Conclusions

This mixed-methods, community-based participatory study demonstrates that the environmental changes affecting the Casamance including deforestation, altered rainfall patterns, soil salinization, declining fish stocks, and the loss or abandonment of arable land are deeply felt, widely recognized, and accurately interpreted by the community members and farmers who experience them daily. Their assessments mirror regional climate and ecological data, confirming that experiential knowledge is an essential and underutilized source of environmental monitoring. Community members not only identified the drivers and consequences of environmental change but also articulated pathways through which these processes undermine their food security, cultural continuity, and land stewardship.

Across focus groups, interviews, and survey data, participants emphasized that these challenges cannot be addressed solely through individual adaptation. Their proposed solutions including reforestation, strengthened environmental protection, improved land management, and expanded mandates for governmental agencies reflect an understanding that meaningful change requires upstream, structural, and policy-level action. The community in this study also highlighted the need to include cultural and spiritual dimensions of land care, particularly the role of sacred forests and the leadership of women in shaping environmental knowledge and conservation practices. In Casamance, ecological degradation is experienced not only as the loss of productive land, but as the erosion of ancestral relationships and community identity. Interventions that do not integrate these cultural frameworks risks misunderstanding both the nature of the problem and the solutions already being enacted locally. As climate change increases the frequency of environmental and socio-economic shocks, investing in local food systems, particularly, those stewarded by women will be essential to strengthening community resilience.

Taken together, this study underscores that responding to climate-related food insecurity in Casamance requires more than technical interventions. It calls for approaches that elevate farmer knowledge, protect culturally significant ecological landscapes, and prioritize policy reforms that address the structural drivers of environmental degradation.

## Data Availability

A partial dataset may be made available upon reasonable request to the senior author and with the approval of the appropriate community partners and ethics committees.
